# Optimal cancer prognosis under network uncertainty

**DOI:** 10.1186/s13637-014-0020-3

**Published:** 2015-01-27

**Authors:** Mohammadmahdi R Yousefi, Lori A Dalton

**Affiliations:** 1 Department of Electrical and Computer Engineering, The Ohio State University, Columbus, 43210 OH USA; 2 Department of Biomedical Informatics, The Ohio State University, Columbus, 43210 OH USA

## Abstract

Typically, a vast amount of experience and data is needed to successfully determine cancer prognosis in the face of (1) the inherent stochasticity of cell dynamics, (2) incomplete knowledge of healthy cell regulation, and (3) the inherent uncertain and evolving nature of cancer progression. There is hope that models of cell regulation could be used to predict disease progression and successful treatment strategies, but there has been little work focusing on the third source of uncertainty above. In this work, we investigate the impact of this kind of network uncertainty in predicting cancer prognosis. In particular, we focus on a scenario in which the precise aberrant regulatory relationships between genes in a patient are unknown, but the patient gene regulatory network is contained in an uncertainty class of possible mutations of some known healthy network. We optimistically assume that the probabilities of these abnormal networks are available, along with the best treatment for each network. Then, given a snapshot of the patient gene activity profile at a single moment in time, we study what can be said regarding the patient’s treatability and prognosis. Our methodology is based on recent developments on optimal control strategies for probabilistic Boolean networks and optimal Bayesian classification. We show that in some circumstances, prognosis prediction may be highly unreliable, even in this optimistic setting with perfect knowledge of healthy biological processes and ideal treatment decisions.

## Introduction

NCI defines cancer prognosis as ‘...an estimate of the likely course and outcome of a disease. The prognosis of a patient diagnosed with cancer is often viewed as the chance that the disease will be treated successfully and that the patient will recover’ [1]. A central problem in translational medicine is thus to decide, given biological knowledge and a collection of observations, whether a cancer patient will bear any chance of successful treatment.

There are a myriad of approaches to model both normal (healthy) and aberrant (cancerous) cell dynamics, including biological pathways, co-expression networks, Bayesian networks, Boolean networks (BNs), probabilistic BNs (PBNs), Petri nets, differential equation-based networks, etc. It is believed that these may be used to predict disease diagnosis, progression, and successful treatment strategies, which has led to much work on the identification and analysis of biological networks in genomics and biomedicine.

There remain two questions regarding prognosis. First, even if the underlying network of a patient were perfectly known and the best drug to use for the patient were also known, would a patient necessarily be curable? Second, suppose the precise network of a patient were unknown, but probabilities of an uncertainty class of networks, for instance all possible mutations of some healthy network, were available along with the best drug to use for each abnormal network. Then based on available measurements, say genomic or proteomic profiles of the patient, what could be said regarding a patient’s treatability and prognosis? That is, might the very nature of cancer, with its uncertain progression and unique characteristics in each individual, make it impossible to predict prognosis, even given perfect knowledge of all biological processes and ideal treatment decisions? In this paper, we give quantitative answers to these questions, at least at a conceptual level in the context of optimal control strategies for PBNs, by studying intervention outcome in a framework of uncertain biology.

PBNs are a class of dynamical models for functional gene regulatory networks (GRNs) [2]. They can capture the intrinsic uncertainty of gene interactions and measurement error, rendering GRN dynamics as Markov chains. They also provide a systematic way of modeling intervention scenarios, where the theory of discrete-time Markov decision processes can be applied to determine optimal intervention strategies. The steady-state distribution (SSD) of the model Markov chain reflects the long-term behavior (phenotypes) of the underlying network, and changes imposed on the SSD through various types of network intervention serve as a guide for developing beneficial treatment strategies. In short, given a PBN, one can optimally design an intervention strategy to alter the dynamics of the network so that the gene activity profiles (GAPs) evolve in a desired manner.

Managing uncertainty is especially important in modeling biological networks, where there is inherent uncertainty in the state of a network due to immeasurable latent variables, as well as uncertainty due to a lack of knowledge or partial knowledge of the relationships between observable variables even in a healthy network [3]. Here, we focus on a third source of model uncertainty due to the inherent unpredictability of somatic gene mutations or aberrant pathway malfunctioning that may arise in a cancer. This corresponds to listing plausible scenarios in which a healthy network may undergo a functional disruption in normal gene regulation. It is imperative to take into account this uncertainty to provide a robust decision regarding cancer prognosis.

We assume that a patient’s network belongs to an uncertainty class of networks, each derived from a known healthy network that contains some structure essentially common to all networks. Each network in the uncertainty class possesses one or more ‘mutations’ of the healthy network, representing various possible subtypes or stages of cancer. Some networks in the uncertainty class may be very treatable (good prognosis), while others may be difficult or impossible to treat (bad prognosis). In fact, we will partition the space of networks into four classes based on the severity of disease with treatment and the benefit of treatment. We measure the severity of disease by the long-run probability that cancerous cells visit certain known undesirable states, or equivalently, the SSD mass of these undesirable states. We measure the benefit of treatment by the difference between steady-state mass in undesirable states before and after treatment, which we call the steady-state shift.

Our objective is to optimally classify patients into our four prognosis categories and to study the impact of network uncertainty on predicting prognosis. Recent work on optimal Bayesian classification (OBC) furnishes an elegant framework for designing optimal classifiers and optimally estimating their error [4,5]. In the general setting, it is assumed that the true underlying sampling distribution belongs to a parameterized uncertainty class of distributions associated with a known prior probability distribution. Closed-form solutions are available for several models with conjugate priors.

In prior work, there have been several studies developing subnetwork markers extracted from protein or gene interaction networks to improve cancer diagnosis [6-9]. While it is clear that classifier performance can be greatly improved using subnetwork markers, these works only consider groups of components known to interact and do not take full advantage of network structure itself. Furthermore, these works focus on diagnosis and do not model the effect of intervention. Work in [10] proposes a competition-based strategy using large datasets to identify the best methods to predict breast cancer prognosis. Several methods are employed using genomic or clinical information or both. While the authors demonstrate that some of the best methods for prognosis prediction incorporate molecular features selected by expert prior knowledge along with both molecular and clinical data, all methods used are based on data-driven machine learning rather than optimal prediction and error estimation and do not take full advantage of network structure to improve prediction. In [11,12], the authors present methods of constructing uncertainty classes of gene expression distributions in the OBC framework that are consistent with available pathway information to improve classification. However, the focus is on diagnosis rather than prognosis, and these works treat network uncertainty as stemming from ignorance. For instance, they assume that all data is drawn from the same sampling distribution, rather than modeling multiple subtypes of cancer that may exhibit different patterns of gene expression. While these advances improve cancer classification using various forms of prior knowledge, no work that we know of rigorously addresses optimal error rates that can be achieved in the presence of uncertain knowledge of the underlying network due to the inherent heterogeneity of cancer.

**Figure 1:**
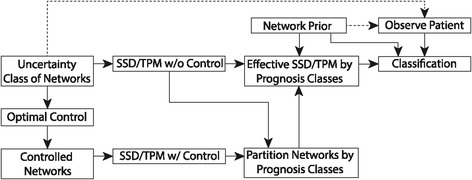
**A schematic of our procedure to study prognosis prediction.**

In this work, we assume a single GAP is observed from the patient, which is essentially a snapshot of the state of the patient’s network at the moment the sample is drawn. The patient’s sampling distribution is thus equivalent to the steady-state distribution of their network without intervention, giving a correspondence between the uncertainty class of networks and the uncertainty class of sampling distributions. We impose a prior distribution over the uncertainty class of networks, with the interpretation that certain mutation events are more or less likely with known probabilities. We can therefore cast our classification problem in a discrete Bayesian setting and directly apply closed-form optimal Bayesian classification and Bayesian error analysis. Note there is no training data *per se*, since we are modeling uncertainty in the progression of cancer itself while assuming a perfect understanding of cell regulation, as opposed to modeling uncertainty due to ignorance of biological relationships between genes, where knowledge could be enriched with training examples. Figure 1 illustrates a schematic of our procedure to study prognosis prediction.

## Network model

Since PBNs fundamentally rely on the dynamics of constituent BNs, we shall define BNs first. A BN is characterized by a set of *n* *nodes*, *v*_*i*_∈{0,1} for *i*=1,…,*n*, representing the expression level of genes or their products, and a collection of *n* Boolean *predictor functions*, *f*_*i*_:{0,1}^*n*^→{0,1} for *i*=1,…,*n*, describing the functional relationships between genes. In this setting, 0 and 1 represent down- and upregulation of genes, respectively.

The GAP is defined to be a length-*n* binary vector, $\mathbf {v}^{k}=\left [{v_{1}^{k}},{v_{2}^{k}},\ldots,{v_{n}^{k}}\right ]$, describing the expression level of all *n* genes at time *k*=0,1,…, where ${v_{i}^{k}} \in \{0, 1\}$ is the value of node *i* at time *k*. The Boolean function *f*_*i*_ determines the value of node *i* at time *k*+1 by $v_{i}^{k+1}=f_{i}\left (\mathbf {v}^{k}\right)$. Although *f*_*i*_ takes as input the entire GAP, **v** ^*k*^, in general it might depend on only *r*(*i*)*predictor nodes* for gene *i*. We assume all genes update synchronously. Several methods for constructing transition rules have been proposed, for instance the ‘majority vote’ rule [3,13,14], and the ‘strong inhibition’ rule [15]. Here, we adopt the former method. For a BN, we define a regulatory matrix, , with (*i*,*j*) component

(1) $$ \mathcal{R}_{ij} = \begin{cases} 1 & \text{if gene} \;j\; \text{activates gene} \;i, \\ -1 & \text{if gene}\; j\; \text{suppresses gene} \;i, \\ 0 & \text{otherwise.} \end{cases}  $$

Therefore, row *i* of  has *r*(*i*) non-zero elements. The majority vote rule stipulates that a gene should become upregulated if more activating genes are ON than suppressing genes, downregulated if more suppressing genes are ON than activating genes, and stay the same otherwise. Thus, we define the regulatory functions

(2) $$ f_{i}\left(\mathbf{v}^{k}\right) = \begin{cases} 1 & \text{if}\,\, \sum_{j} \mathcal{R}_{ij} {v_{j}^{k}} > 0, \\ 0 & \text{if} \,\,\sum_{j} \mathcal{R}_{ij} {v_{j}^{k}} < 0, \\ {v_{i}^{k}} & \text{otherwise.} \end{cases}  $$

There is a natural bijection between **v** ^*k*^ and its integer representation $x^{k}\in \mathcal {S}=\{0,1,\ldots,2^{n}-1\}$ given by $x^{k}=\sum _{i=1}^{n} 2^{n-i} {v_{i}^{k}}$. We call *x* ^*k*^ the *state* of the network at time *k* and  the *state space*.

PBNs generalize BNs by introducing random switching between several *contexts*, where each context is a BN on its own. They also introduce a random gene perturbation, where the current state of each gene in the network is randomly flipped with probability *p*. If the PBN has only a single context, then the model becomes a BN with perturbation (BNp), which will serve as our model for GRNs in this paper.

Probabilistic transition rules of any PBN can be modeled by a homogeneous Markov chain. We denote the stochastic process of state transitions by $\{Z^{k}\in \mathcal {S} : k=0,1,\ldots \}$. Originating from state $x\in \mathcal {S}$, the successor state $y\in \mathcal {S}$ is selected according to the transition probability matrix (TPM) , with (*x*,*y*) element $\mathcal {P}_{\textit {xy}}:= P(Z^{k+1}=y\mid Z^{k}=x)$ for all *k*=0,1,… [2]. Due to random gene perturbation, the equivalent Markov chain is ergodic and has a unique invariant distribution, *π*, equal to the SSD of the network under no intervention. We also use *π*_*x*_ to denote the probability mass of *π* evaluated at state $x \in \mathcal {S}$.

## Optimal intervention in PBNs

Treatment aims to alter the dynamics of a cell to achieve some desirable property or behavior. To formalize this for a given PBN, let  be a set of *undesirable* states, which may be an arbitrary subset of . States in  may correspond to pathological behavior or known cancer phenotypes. A natural measure of the performance of a treatment or control policy then becomes the long-run expected occupation of undesirable states. We now review optimal intervention, assuming the true TPM is perfectly known.

Two types of intervention methods for PBNs have been proposed: *structural intervention* [16] and *external control* [17,18]. The former aims to effectively change the wiring of a GRN so that long-run dynamics of the underlying Markov chain are moved toward beneficial states. Several advanced techniques, such as siRNA interference, can carry out pathway blockage [3]. The latter method involves designing a program for taking actions over time that alter the expression level of some genes (or gene products), known as *control genes*, effectively steering the long-run dynamics of the network away from undesirable states. This type of intervention corresponds to intervention using drugs to act on gene products. In this paper, we choose the latter method and assume that the PBN admits an external control input *a* from a set of actions, $\mathcal {A}=\{0,1\}$, where *a*=0 indicates no-intervention and *a*=1 indicates that the expression level of a single control gene, corresponding to a node *c*∈{1,2,…,*n*}, is flipped. Under control action *a*=1, the transition probabilities at state *x*, or equivalently the row corresponding to *x* in the original TPM, are replaced by the row corresponding to state $\tilde {x}$ having the same binary representation as *x* except with node *v*_*c*_ flipped. Let $\left \{\left (Z^{k}, A^{k}\right) \in \mathcal {S} \times \mathcal {A} : k = 0, 1, \ldots \right \}$ denote the stochastic process of states and actions taken. The transition rules for the controlled PBN are given by a new TPM, $\mathcal {P}(a)$, with (*x*,*y*) element $\mathcal {P}_{\textit {xy}}(a)=P(Z^{k+1}=y\mid Z^{k}=x, A^{k}=a)$, for *k*=0,1,…. The ergodicity of the controlled TPM, $\mathcal {P}(a)$, for each $a \in \mathcal {A}$, is immediate from the ergodicity of the original uncontrolled TPM, .

Suppose we wish to optimally steer the dynamics away from undesirable states by applying a regimen of external control actions at each time *k*=0,1,…,*N*. This optimization problem has been well-studied in the context of optimal Markov decision processes. We define a *control policy*, *μ*={*μ* ^0^,*μ* ^1^,…,*μ* ^*N*^}, as a sequence of instructions for taking actions that take into account the entire history of states and actions up to time *k*, *h* ^*k*^=(*z* ^0^,*a* ^0^,*z* ^1^,*a* ^1^,…,*z* ^*k*^,*a* ^*k*^). In particular, after observing the history, *h* ^*k*−1^, and the current state, *z* ^*k*^, the control policy prescribes action $a\in \mathcal {A}$ with some designated probability *μ* ^*k*^(*a*∣*h* ^*k*−1^,*z* ^*k*^), satisfying 0≤*μ* ^*k*^(*a*∣*h* ^*k*−1^,*z* ^*k*^)≤1 and $\sum _{a\in \mathcal {A}} \mu ^{k} \left (a\mid h^{k-1}, z^{k}\right)=1$.

Denote the class of all control policies by . Two classes of policies of particular interest are *stationary randomized* and *stationary deterministic* policies, denoted by $\mathcal {M}_{\text {SR}}$ and $\mathcal {M}_{\text {SD}}$, respectively. $\mathcal {M}_{\text {SR}}$ includes policies that are time invariant, where *μ* ^*k*^ does not depend on *k* and is only conditioned on the current state. $\mathcal {M}_{\text {SD}}$ is a subset of $\mathcal {M}_{\text {SR}}$ and defined to be the set of all stationary deterministic policies such that *μ* ^*k*^ is either 0 or 1, depending on *a*, for every state in . In this case, the control policy is a deterministic function from  to . Given the initial state *Z* ^0^=*x* of the Markov chain and any policy *μ*, one can determine a unique probability measure $\mathrm {P}_{x}^{\mu }$ over the space of all trajectories of states and actions, which correspondingly defines the joint stochastic processes (*Z* ^*k*^,*A* ^*k*^) of the states and actions for the controlled system [19]. Let **1**_*A*_ denote an indicator function, where **1**_*A*_(*x*) is one if *x*∈*A* and zero otherwise. Our goal is to minimize the long-run expected occupation of undesirable states or equivalently to minimize the objective

(3) $$\begin{array}{*{20}l} J(x,\mu) = \limsup_{N\rightarrow \infty} \mathrm{E}_{x}^{\mu} \left[ \frac{1}{N+1} \sum_{k=0}^{N} \mathbf{1}_{\mathcal{U}} \left(Z^{k}\right) \right], \end{array} $$

where $\mathrm {E}_{x}^{\mu }$ denotes the expectation relative to $\mathrm {P}_{x}^{\mu }$ [20]. Let $J^{\ast }(x) = \inf _{\mu \in \mathcal {M}}~J(x, \mu)$ for any initial state $x \in \mathcal {S}$. A policy *μ* ^∗^ is optimal if *J* ^∗^(*x*)=*J*(*x*,*μ* ^∗^), for every $x\in \mathcal {S}$. It can be shown that there exists an optimal control policy that belongs to $\mathcal {M}_{\text {SD}}$, and that *J* ^∗^(*x*) is independent of the initial state *x* [19].

While this optimization problem can be solved with dynamic programming, it may also be formulated as a classical linear program (LP) that minimizes the long-run expected frequency of undesirable states and control action pair for policies in $\mathcal {M}_{\text {SR}}$ [19,21,22]. The LP formulation, reviewed in the remainder of this section, requires that for any $\mu \in \mathcal {M}_{\text {SD}}$, the underlying Markov chain be ergodic, which holds true for PBNs. Given a family of TPMs, $\mathcal {P}(a)$ for $a \in \mathcal {A}$, and any policy $\mu \in \mathcal {M}_{\text {SR}}$, we can obtain the TPM of the controlled process, $\mathcal {Q}(\mu)$, via

(4) $$\begin{array}{*{20}l}  \mathcal{Q}_{xy}(\mu)=\sum_{a\in \mathcal{A}} \mathcal{P}_{xy}(a)\mu (a|x), \end{array} $$

where $\mathcal {Q}_{\textit {xy}}(\mu)$ is the (*x*,*y*) element of $\mathcal {Q}(\mu)$, and *μ*(*a*|*x*) is the probability distribution on actions prescribed by *μ* given the current state. Let $\pi (\mu) = [\pi _{0} (\mu), \pi _{1} (\mu), \ldots, \pi _{|\mathcal {S}|-1}(\mu)]$ denote the unique invariant vector of $\mathcal {Q}(\mu)$ such that, for all $x\in \mathcal {S}$,

(5) $${} \pi (\mu) = \pi (\mu)\mathcal{Q}(\mu), \quad 0 \leq \pi_{x} (\mu) \leq 1, \quad \displaystyle\sum_{x\in \mathcal{S}} \pi_{x}(\mu) = 1.  $$

The joint probability mass of any state-action pair, *x* and *a*, as a function of *μ* is defined by *ν*_*xa*_(*μ*):=*μ*(*a*|*x*)*π*_*x*_(*μ*), where we have ${\sum _{a\in \mathcal {A}} \nu _{\textit {xa}}(\mu) = \pi _{x} (\mu)}$. Then, it can be shown that for any $x \in \mathcal {S}$, *J*(*x*,*μ*)=*J*(*μ*), where

(6) $$\begin{array}{*{20}l} {J(\mu) = \sum_{x\in \mathcal{U}} \sum_{a\in \mathcal{A}} \nu_{xa}(\mu)}. \end{array} $$

Hence, the original optimization problem can be reduced to the following LP:

$$\begin{array}{ll} & {\displaystyle\min_{\{\nu_{xa}\}} \sum_{x\in \mathcal{U}} \sum_{a\in \mathcal{A}}} \nu_{xa}, \vspace{5pt} \\ & \text{subject to~} \left\{ \begin{array}{l} {\displaystyle\sum_{a\in \mathcal{A}}}\nu_{xa} = {\displaystyle\sum_{y\in \mathcal{S}} \sum_{a\in \mathcal{A}}} \nu_{ya} \mathcal{P}_{yx}(a), \forall x \in \mathcal{S}, \vspace{5pt} \\ {\displaystyle\sum_{x\in \mathcal{S}} \sum_{a\in \mathcal{A}}} \nu_{xa} = 1, \nu_{xa}\geq 0, \forall x\in \mathcal{S}, a\in\mathcal{A}. \end{array} \right. \end{array} $$

Let $\{\nu ^{\ast }_{\textit {xa}}\}$ be minimizing arguments of the above problem. Then, an optimal policy $\mu ^{\ast } \in \mathcal {M}_{\text {SR}}$ is given by:

(7) $$ \mu^{\ast}(a|x) = \frac{\nu_{xa}^{\ast}}{{\displaystyle\sum_{a\in \mathcal{A}}} \nu_{xa}^{\ast}}.   $$

Although the search space for *μ* is $\mathcal {M}_{\text {SR}}$, it can be shown that ${\mu ^{\ast } \in \mathcal {M}_{\text {SD}}}$ [19,21,22]. Furthermore, since the controlled Markov chain is ergodic, ${\sum _{a\in \mathcal {A}}} \nu _{\textit {xa}}^{\ast } \neq 0$ for all $x\in \mathcal {S}$.

## Network uncertainty class

Having established a method to model networks and optimal intervention, we next discuss a model for network uncertainty that captures variability among cancer patients due to unpredictable and compounding mutations. Essentially, we assume that the patient’s network belongs to an uncertainty class of possible ‘cancer’ networks that are the result of one or several detrimental modifications (mutations) of a nominal ‘healthy’ network.

Let $\mathcal {R}^{H}$ denote the regulatory matrix of a nominal healthy network, which possesses a small steady-state mass in undesirable states. We denote our uncertainty set of regulatory matrices by *Θ* and impose two constraints: (1) regulatory matrices in *Θ* differ from $\mathcal {R}^{H}$ by only a few number of elements. For example, assuming that each mutation, or perturbation, corresponds to a random edge addition (0 is mutated to 1 or −1) or removal (1 or −1 is mutated to 0), each element in *Θ* might have up to some number of edges added or removed relative to $\mathcal {R}^{H}$. We allow different limits to the number of edges added versus removed, but assume that the total number of each type of edge mutation in any regulatory matrix of *Θ* is small relative to the size of the network. (2) *Θ* should contain only regulatory matrices for which the undesirable steady-state mass is greater than some threshold. Thus, cancers in our model have detrimental effects as mutations accumulate.

To reflect the reality that cancer cells with more mutations are more rare and that certain types of perturbations may be more or less likely, we assign prior probabilities to every network represented in *Θ*. To this end, we assume that the number of mutations of a network in *Θ* follows essentially a truncated geometric distribution, where the probability of *l* mutations is proportional to *γ* ^*l*^ for some 0<*γ*≤1 (normalization is necessary since the number of mutations of networks in *Θ* is bounded). We further assume that all networks with *l* mutations are equally likely, for example, if there are *N*_*l*_ regulatory matrices in *Θ* that have *l* elements mutated with respect to $\mathcal {R}^{H}$, then they are all equally likely with probability proportional to *γ* ^*l*^/*N*_*l*_. Once we have calculated these values for all elements of *Θ*, we normalize their sum to one, guaranteeing a valid probability distribution, and denote the resulting probability distribution by *Λ*, i.e., we have $\sum _{\mathcal {R} \in \Theta } \Lambda (\mathcal {R}) = 1$ and $\Lambda (\mathcal {R}) > 0$ for all $\mathcal {R} \in \Theta $.

Each  in *Θ* induces a SSD under no intervention, which we denote by $\pi _{\mathcal {R}}$. Also, let $\pi _{\mathcal {R}x}$ be the SSD of  evaluated at point $x \in \mathcal {S}$, and let $\Pi = \{\pi _{\mathcal {R}} : \mathcal {R} \in \Theta \}$ be the multiset of all SSDs corresponding to networks in *Θ*. Note that SSDs in *Π* may not be unique.

Each  is also associated with an optimal control policy $\mu ^{\ast }_{\mathcal {R}} \in \mathcal {M}_{\text {SD}}$ resulting in a new optimally controlled network, $\mathcal {R}^{\ast }$, with SSD $\pi _{\mathcal {R}^{\ast }}$ having minimal undesirable steady-state mass with respect to all control policies. Note that in general, every control policy $\mu \in \mathcal {M}_{\text {SR}}$ induces a controlled network, $\mathcal {R}_{\mu }$, for every $\mathcal {R} \in \Theta $. We can partition *Θ* into several sets based on intervention results. For example, one might calculate the steady-state mass of undesirable states after intervention and label the outcome with either a ‘good’ (low undesirable mass) or ‘poor’ (high undesirable mass) prognosis. One may also be interested in whether it is worth intervening in the sense that the steady-state mass of undesirable states shifts substantially with optimal control. Hence, we partition *Θ* into four prognosis classes:

- Class 1 (*Θ* ^1^): $\pi _{\mathcal {R}^{\ast }\mathcal {U}} < \alpha $ and $\pi _{\mathcal {R}\mathcal {U}} - \pi _{\mathcal {R}^{\ast }\mathcal {U}} < \beta _{1}$ (patient’s condition is not critical),
- Class 2 (*Θ* ^2^): $\pi _{\mathcal {R}^{\ast }\mathcal {U}} < \alpha $ and $\pi _{\mathcal {R}\mathcal {U}} - \pi _{\mathcal {R}^{\ast }\mathcal {U}} \geq \beta _{1}$ (patient responds well to an effective treatment),
- Class 3 (*Θ* ^3^): $\pi _{\mathcal {R}^{\ast }\mathcal {U}} \geq \alpha $ and $\pi _{\mathcal {R}\mathcal {U}} - \pi _{\mathcal {R}^{\ast }\mathcal {U}} \geq \beta _{2}$ (patient’s condition can be improved to some extent),
- Class 4 (*Θ* ^4^): $\pi _{\mathcal {R}^{\ast }\mathcal {U}} \geq \alpha $ and $\pi _{\mathcal {R}\mathcal {U}} - \pi _{\mathcal {R}^{\ast }\mathcal {U}} < \beta _{2}$ (patient’s condition is poor and cannot be improved).

Here, $\pi _{\mathcal {R}\mathcal {U}} = \sum _{x \in \mathcal {U}} \pi _{\mathcal {R}x}$ and $\pi _{\mathcal {R}^{\ast }\mathcal {U}} = \sum _{x \in \mathcal {U}} \pi _{\mathcal {R}^{\ast }x}$ denote the accumulated steady-state mass of undesirable states under no intervention and optimal intervention, respectively, and 0<*α*,*β*_1_,*β*_2_≤1. Further, note the probability that a network belongs to *Θ* ^*i*^ is given by

(8) $$\begin{array}{@{}rcl@{}}</p><p class="noindent">c^{i} = \sum_{\mathcal{R}' \in \Theta^{i}} \Lambda\left(\mathcal{R}'\right) \end{array} $$

and define the probability distribution *Λ* ^*i*^ to be the conditional probability of the networks in *Θ* ^*i*^:

(9) $$  \Lambda^{i}(\mathcal{R}) = \frac{\Lambda(\mathcal{R})}{c^{i}}  $$

for every $\mathcal {R} \in \Theta ^{i}$ and *i*∈{1,2,3,4}.

## Bayesian classification

Our objective is now to study optimal classification of patients into the four prognosis classes. A classifier, *ψ*, is a function that takes as input observations, in our case a point $x \in \mathcal {S}$ representing the GAP of a cancer patient at a single time epoch, and outputs a prediction of some unknown label associated with the observations, here a member of {1,2,3,4} representing one of four possible prognoses of the patient. In general, classification performance depends on the underlying sampling distribution governing observations, which in our model is precisely the steady-state distribution of the patient’s network without control. Were the network of the patient perfectly known, prognosis could be determined perfectly as the class corresponding to this network, and it would not be necessary to obtain a GAP for the patient. In the case of network uncertainty, prediction is no longer perfect and observing the GAP of a patient potentially aids in making a better prognosis.

To perform optimal classification, we utilize OBC theory, which is founded on a Bayesian framework that models uncertainty in the underlying sampling distributions [4,5]. Essentially, a prior probability is assigned to all sampling distributions in an uncertainty class that may have produced the observed sample. In our application, the prior probability on the uncertainty class of networks induces a prior on the uncertainty class of steady-state distributions without control, making OBC classification very natural to implement. The main idea is to leverage minimum mean-square error (MMSE) estimation theory to obtain an optimal Bayesian error estimates for any classifier. Thanks to MMSE estimation theory, the optimal Bayesian error estimate (BEE) is precisely the expected misclassification rate with respect to the prior. The optimal Bayesian classifier is then defined to be that classifier which minimizes the BEE.

In the usual implementation of OBC, uncertainty is interpreted as more of an issue of ignorance, where there are some true underlying class-conditional distributions, but their identity in the uncertainty class is unknown and can be revealed with training data. Here, all distributions in the uncertainty class may exist in the population, and the issue is in devising a robust classifier that can be applied generally to all distributions in the uncertainty class with minimal expected error. A consequence is that training data from different patients generally cannot be used to collapse the prior to a tighter posterior, unless care is taken to consider known connections to the patient of interest.

Given a specific network in $\mathcal {R} \in \Theta $ having label *i* and sampling distribution $\pi _{\mathcal {R}} \in \Pi $, and an arbitrary classifier *ψ*, let $\varepsilon _{\mathcal {R}} (\psi)$ denote the misclassification rate of *ψ* under $\pi _{\mathcal {R}}$:

(10) $$ \varepsilon_{\mathcal{R}} (\psi) = \sum_{x : \psi(x) \neq i} \pi_{\mathcal{R} x}.  $$

Now, suppose  is unknown. Let $\mathcal {L} = \{1,2,\ldots, L\}$, where *L* is the number of classes, each associated with a set of networks *Θ* ^*i*^, a multiset of sampling distributions, $\{\pi _{\mathcal {R}} : \mathcal {R} \in \Theta ^{i}\}$, and priors, $\{\Lambda ^{i}(\mathcal {R}) : \mathcal {R} \in \Theta ^{i}\}$. A natural metric for classifier performance is the expected misclassification rate, $\hat {\varepsilon }(\psi) = \mathrm {E}_{\Lambda } [\!\varepsilon _{\mathcal {R}} (\psi)]$, where E_*Λ*_ denotes an expectation over  with respect to the distribution *Λ*. One can show that $\hat {\varepsilon }(\psi) = \sum _{i \in \mathcal {L}} c^{i} \mathrm {E}_{\Lambda ^{i}} [\!\varepsilon _{\mathcal {R}} (\psi)]$, where $\mathrm {E}_{\Lambda ^{i}}\phantom {\dot {i}\!}$ denotes an expectation over $\mathcal {R} \in \Theta ^{i}$ with respect to conditional distribution *Λ* ^*i*^. This quantity is, in fact, equivalent to the BEE, where the class probabilities, *c* ^*i*^, are perfectly known, and $\mathrm {E}_{\Lambda ^{i}} [\!\varepsilon _{\mathcal {R}} (\psi)]$ is the expected error contributed by class *i*.

The OBC formalized in [4] is defined by

(11) $$\begin{array}{@{}rcl@{}}  \psi_{\text{OBC}} = \arg \inf_{\psi \in \mathcal{C}} \mathrm{E}_{\Lambda} \left[\varepsilon_{\mathcal{R}} (\psi)\right], \end{array} $$

where  is the space of all classifiers. For every $i \in \mathcal {L}$, we define the *effective density* at point $x \in \mathcal {S}$ by

(12) $$\begin{array}{@{}rcl@{}}  {f^{i}_{x}} = \sum_{\mathcal{R} \in \Theta^{i}} \pi_{\mathcal{R}x} \Lambda^{i}(\mathcal{R}). \end{array} $$

The following theorem shows how *ψ*_OBC_ can be found [4].

### Theorem 1.

An optimal Bayesian classifier, *ψ*_OBC_, satisfying Equation 11 exists and at point $x \in \mathcal {S}$ is given by *ψ*_OBC_(*x*)=*i*, where $i \in \mathcal {L}$ is such that $c^{i} {f^{i}_{x}} \geq c^{j} {f^{j}_{x}}$ for all $j \in \mathcal {L}$. In the event of a tie, by convention we choose the class, *i*, satisfying $c^{i} {f^{i}_{x}} \geq c^{j} {f^{j}_{x}}$ for all $j \in \mathcal {L}$ with the smallest index.

Using Equations 9 and 12, we can rewrite the above condition and assign $x \in \mathcal {S}$ to class $i \in \mathcal {L}$ if

(13) $$\begin{array}{@{}rcl@{}}  \sum_{\mathcal{R} \in \Theta^{i}} \pi_{\mathcal{R}x} \Lambda(\mathcal{R}) \geq \sum_{\mathcal{R} \in \Theta^{j}} \pi_{\mathcal{R}x} \Lambda(\mathcal{R}) \end{array} $$

for all $j \in \mathcal {L}$. The expected misclassification rate of *ψ*_OBC_ is

(14) $$\begin{array}{@{}rcl@{}}  \hat{\varepsilon}(\psi_{\text{OBC}}) = \sum_{x \in \mathcal{S}} \sum_{\substack{j \in \mathcal{L} \\ j \neq \psi_{\text{OBC}}(x)}} \sum_{\mathcal{R} \in \Theta^{j}} \pi_{\mathcal{R}x} \Lambda(\mathcal{R}). \end{array} $$

Furthermore, the probability of label *i* conditioned on a fixed observation $x \in \mathcal {S}$ is given by

(15) $$  \frac{c^{i} {f_{x}^{i}}}{\sum_{j \in \mathcal{L}} c^{j} {f_{x}^{j}}} = \frac{\sum_{\mathcal{R} \in \Theta^{i}} \pi_{\mathcal{R}x} \Lambda(\mathcal{R})}{\sum_{\mathcal{R} \in \Theta} \pi_{\mathcal{R}x} \Lambda(\mathcal{R})},  $$

for $i \in \mathcal {L}$. Whereas Equation 14 evaluates the overall error rate over random networks and observations, Equation 15 may be used to evaluate the error rate over random networks conditioned on a particular observation, *x*.

## Simulation results

In this section, we implement our procedure to study prognosis prediction on synthetically generated networks, as well as two real networks derived from biological processes related to cancer development. The first real network models the mammalian cell cycle, and the second emulates cell response to various stress signals such as DNA damage, oxidative stress, and activated oncogenes.

### Synthetic networks

To construct synthetic uncertainty classes of networks, we begin by outlining a methodology to construct healthy networks that are calibrated to have low undesirable steady-state mass. We generate a *seed regulatory matrix*, $\mathcal {R}^{S}$, by randomly filling each row of $\mathcal {R}^{S}$ with −1 or 1 as follows. Let *r*_max_ denote the maximum number of predictors for each gene. We draw the number of predictors for gene *i*, *r*(*i*), uniformly from the set {1,…,*r*_max_}. The location of the *r*(*i*) non-zero elements in the *i*th row of $\mathcal {R}^{S}$, designating the predictors of gene *i*, are determined by drawing uniformly from the set {*T*⊂{1,2,…,*n*}:|*T*|=*r*(*i*)}. Once the predictors of each gene are determined, we assign 1 to each corresponding location in $\mathcal {R}^{S}$ with probability *β*∈[0,1] and −1 with probability 1−*β*. *β* reflects a bias toward what type of regulatory relationship (activation or suppression) is more likely to occur. Given the perturbation probability *p*, we calculate a TPM and its SSD for the network corresponding to the seed regulatory matrix [23]. We then select a nominal healthy network, $\mathcal {R}^{H}$, as the network with minimum undesirable steady-state mass among all possible networks with a single mutation relative to $\mathcal {R}^{S}$.

Let REM and ADD be two non-negative integers. We enumerate all regulatory matrices such that no greater than REM and ADD edges are removed from or added to $\mathcal {R}^{H}$, respectively. We then exclude networks that have lower undesirable steady-state mass than the healthy network, as well as networks with undesirable steady-state mass less than the average undesirable mass of all networks with single mutations. This guarantees that the set *Θ* contains only networks with unfavorable steady-state distributions. Given *γ*, we then calculate the probability distribution *Λ* for elements of *Θ*.

We generate 250 random seed networks with seven genes (*n*=7). For each network, we select at most three predictors for each gene (*r*_max_=3), with both types of edges being equally likely (*β*=0.5) and set the BNp random gene perturbation probability *p* to 0.01. We define the set of undesirable states, , to be the set of all states in which the gene corresponding to the most significant bit (*v*_1_) in the binary representation of the state is downregulated. This results in half of the states being undesirable. We also set the number of edge removals to REM=1, the number of edge additions to ADD=1, and the mutation probability *γ* to 0.5. Each seed network corresponds to an uncertainty set *Θ*.

In the next stage of our procedure, given a control gene, we design the optimal intervention policy for each $\mathcal {R} \in \Theta $, which results in a controlled SSD $\pi _{\mathcal {R}^{\ast }}$. In our classification settings, *L*=4 and we partition *Θ* into four subsets by choosing *α*, *β*_1_, and *β*_2_ such that these subsets have (almost) equal sizes. Given *Λ*, the prior probability of networks in *Θ*, we use Equation 13 to find the OBC for the uncertainty set *Θ* and probability distribution *Λ*. We also estimate the error of this classifier using Equation 14. Changing the control gene does not affect *Θ*, however it will change the partitioning of *Θ* and classification results. Thus, we set the control gene, in turn, to every gene in the network excluding the target gene.

**Figure 2:**
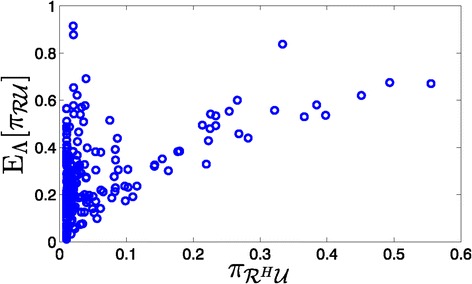
**Expected undesirable steady-state mass of networks without intervention versus the undesirable steady-state mass of** $\boldsymbol {\boldsymbol{\mathcal {R}}^{H}}$ **.**

**Figure 3:**
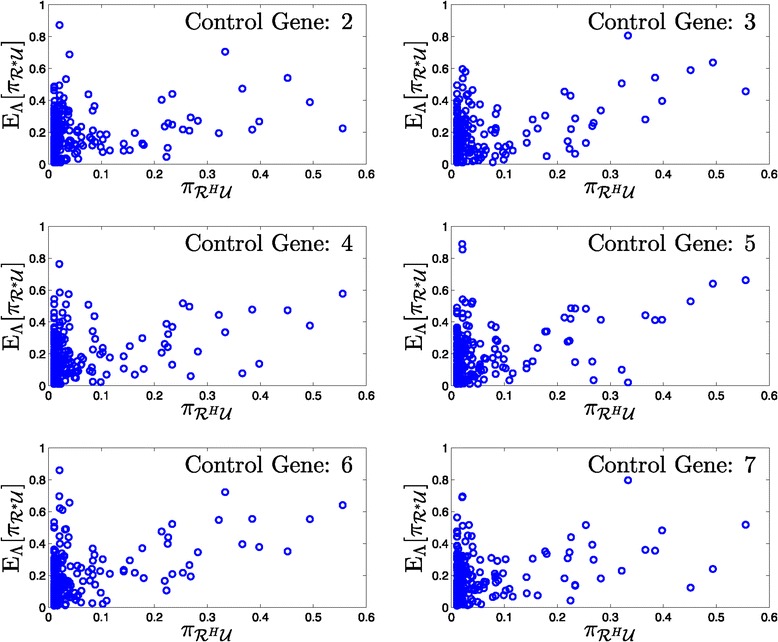
**Expected undesirable steady-state mass of networks after optimal intervention versus the undesirable steady-state mass of** $\boldsymbol {\boldsymbol{\mathcal {R}}^{H}}$ **.**

Figures 2 and 3 show the relationship between the undesirable steady-state mass in the healthy network and the expected (relative to *Λ*) undesirable mass of networks before and after optimal intervention, respectively. In each scatter plot, each point represents a specific seed network and its corresponding uncertainty class. In general, we observe smaller undesirable mass after control, which is not unexpected since, by definition of the objective function, the undesirable mass after applying the optimal control cannot exceed that of the uncontrolled network.

**Figure 4:**
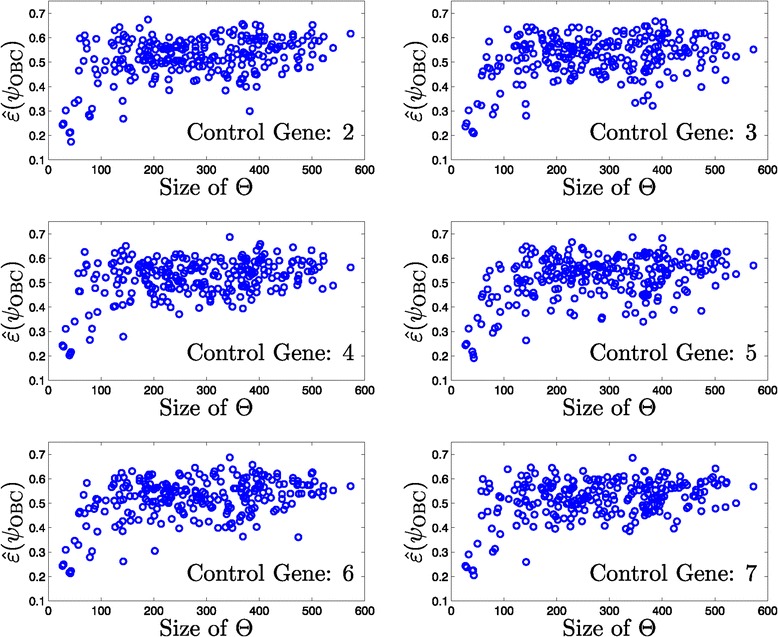
**OBC error rate versus the size of the uncertainty set,** ***Θ*** **.**

Since the seed networks are randomly generated, they differ in the total number of edges and thus produce different sized uncertainty sets, *Θ*. Furthermore, the size of *Θ* is affected by the steady-state criteria for including mutated networks in the set. We expect that as uncertainty regarding the underlying mechanism of cancer increases, i.e., as the size of *Θ* increases, classification becomes a harder task. This effect is observed in Figure 4, where we show scatter plots with respect to the OBC classifier error rate (vertical axis) and the size of uncertainty set *Θ* (horizontal axis). As uncertainty sets grow in size, we observe a trend of increasing error rates.

For a fixed network having label *i*, consider the probability of correct classification with respect to random observed states:

(16) $$ \sum_{x \in \mathcal{S} : \psi_{\text{OBC}}(x) = i} \pi_{\mathcal{R}x}.  $$

**Figure 5:**
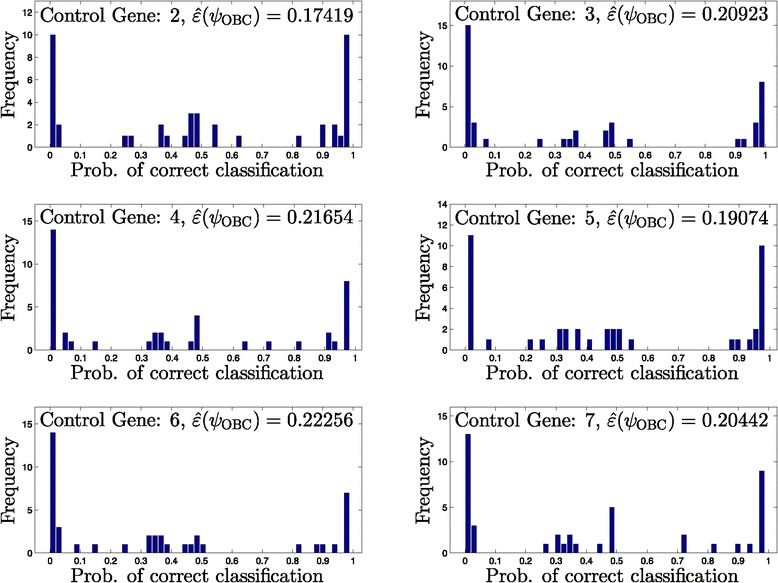
**Histograms of the probability of correctly classifying networks for an uncertainty class.** Low error rate (43 networks in *Θ*).

**Figure 6:**
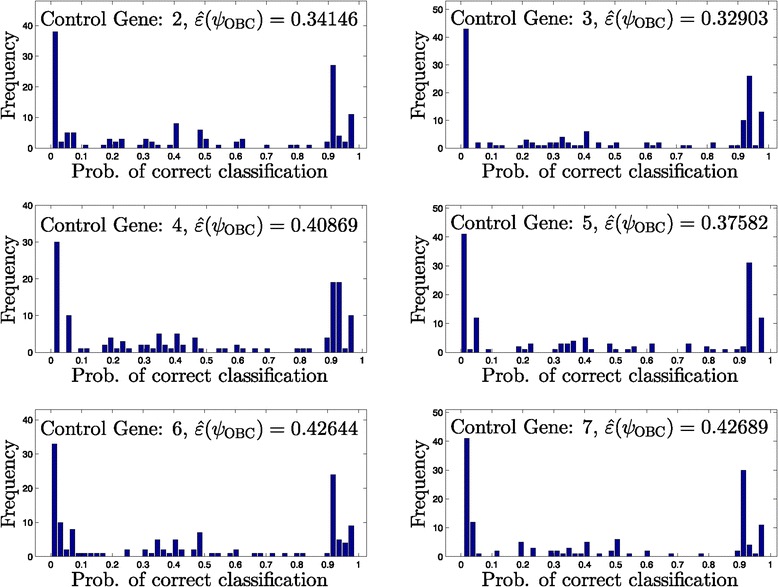
**Histograms of the probability of correctly classifying networks for an uncertainty class.** Moderately low error rate (141 networks in *Θ*).

**Figure 7:**
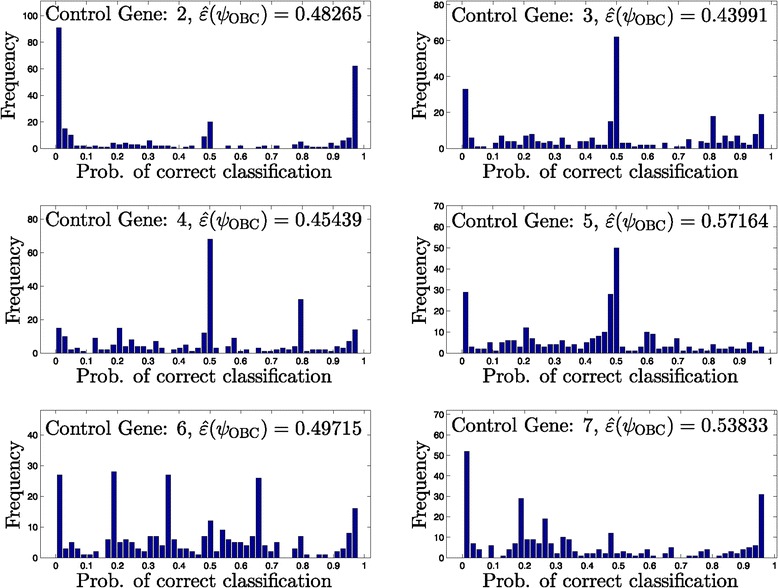
**Histograms of the probability of correctly classifying networks for an uncertainty class.** Moderately high error rate (293 networks in *Θ*).

**Figure 8:**
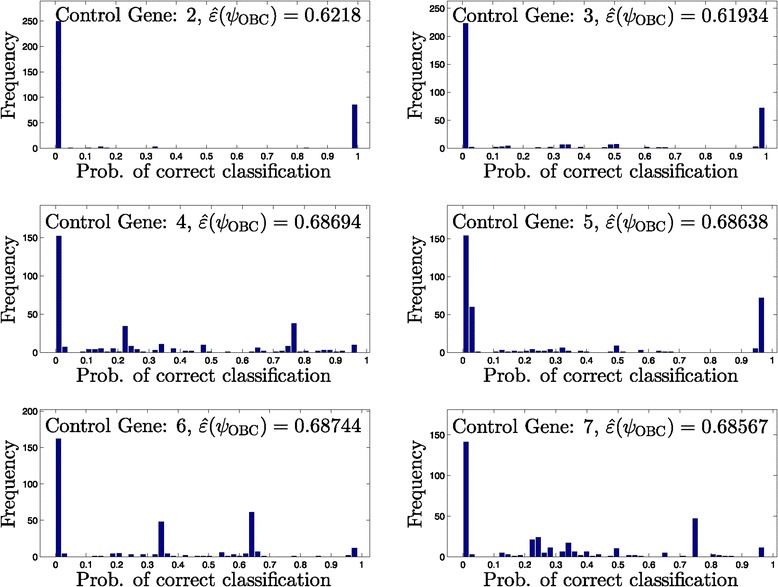
**Histograms of the probability of correctly classifying networks for an uncertainty class.** High error rate (344 networks in *Θ*).

Figures 5, 6, 7 and 8 provide histograms of this probability across all networks in a given uncertainty class. Each figure corresponds to a different uncertainty class (each generated from different seed regulatory matrix) with classification errors at different ranges from low to high, and results under all possible control genes are shown. In almost all cases, the probability of correct classification depends highly on the specific network, for example, in Figure 5, control gene 2, we observe 10 networks out of 43 with nearly zero probability of correct classification, along with 10 networks with nearly perfect classification.

**Figure 9:**
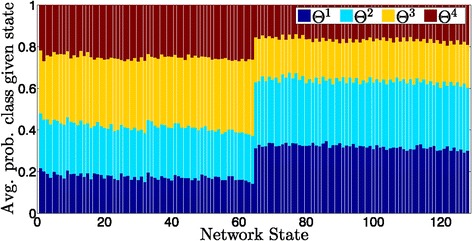
**Conditional probability of each class given the observed state averaged over all 250 classification tasks.** Control gene 7.

For each uncertainty set, class probabilities conditioned on each state may be found across all networks via Equation 15. Figure 9 illustrates the average of these probabilities over all 250 uncertainty sets under control gene 7. By convention, undesirable states are on the left (states 0 through 63). If we observe an undesirable state from the patient, we will most likely classify the patient as class 3 (improvement to some extent), otherwise the classification outcome is most likely class 1, which implies that the patient’s condition is not that critical. However, this figure reflects only an average trend, and results vary considerably for a particular state and uncertainty set.

See the supplementary materials for analogous results on 54 different settings, varying *β*∈{0.1,0.5,0.9}, *p*∈{0.1,0.5,0.9}, *γ*∈{0.005,0.01,0.1}, and *r*_max_∈{2,3}, and a discussion on the effect of these parameters.

### Real networks

#### Mammalian cell-cycle network

We now apply our methodology to a dynamical network modeling the behavior of normal mammalian cells during the cell cycle [24]. The network has ten genes, CycD, Rb, p27, E2F, CycE, CycA, Cdc20, Cdh1, UbcH10, and CycB. Regulatory relationships between genes in this network are shown in Table 1. Three key genes are cyclin D (CycD), retinoblastoma (Rb), and p27. Under normal conditions, extracellular signals, which control the activation of CycD, coordinate cell division with overall growth. The tumor-suppressor gene Rb is expressed in the absence of the cyclins. When present, however, cyclins inhibit Rb by phosphorylation. The gene p27 is also active in the absence of the cyclins. An active p27 blocks the action of CycE or CycA and, hence, Rb can also be expressed even if CycE or CycA are present. This results in a mechanism that stops uncontrolled cell division. However, undesirable cell proliferation in the absence of any growth factor might arise if CycD, Rd, and p27 are all simultaneously downregulated. Therefore, we define the states corresponding to this condition as undesirable states.

**Table 1:** **Regulatory relationships of the mammalian cell cycle network**

**Gene**	**Predictors: regulatory type (+ / −)**
CycD	CycD: +
Rb	CycD: −, p27: +, CycE: −, CycA: −, CycB: −
p27	CycD: −, p27: +, CycE: −, CycA: −, CycB: −
E2F	Rb: −, p27: +, CycA: −, CycB: −
CycE	Rb: −, p27: +, E2F: +, CycE: −, CycA: −
CycA	Rb: −, E2F: +, CycA: +, Cdc20: −,
	Cdh1: −, UbcH10: −
Cdc20	Cdh1: −, CycB: +
Cdh1	p27: +, CycA: −, Cdc20: +, CycB: −
UbcH10	CycA: +, Cdc20: +, Cdh1: −,
	UbcH10: +, CycB: +
CycB	Cdc20: −, Cdh1: −

We construct a BNp following the majority-voting updating rule for this network and set *p*=0.01. This network will serve as the nominal healthy network in our analysis. Due to computational constraints, we only consider regulatory networks for which no more than one edge is removed from $\mathcal {R}^{H}$ and also exclude those having lower undesirable steady-state mass than the healthy network or the average undesirable mass of all networks with a single edge mutation. The set of mutated networks constitutes *Θ*, and since we only allow one mutation, the distribution *Λ* will be uniform. For every network in *Θ*, we take each gene in the network, excluding CycD, Rb, and p27 as control genes in turn, and find the optimal intervention, which maximally shifts the SSD away from undesirable states. Given *Λ*, *Θ*, a control gene and the controlled networks, we follow a similar procedure for partitioning *Θ* as used for the synthetic networks and design an OBC. The results are presented in Table 2 for each control gene, where $\pi _{\mathcal {R}^{H} \mathcal {U}} = 0.3405$, $\mathrm {E}_{\Lambda } [\!\pi _{\mathcal {R}\mathcal {U}}] = 0.3541$ and *Θ* contains 21 mutated networks. Although the average improvement in the SSD of undesirable states is significant, the classification error rates are poor, which indicates that prognosis classification is difficult for this network. The best classification performance in achieved when E2F is the control gene, which is slightly better than random guessing.

**Table 2:** **The expected undesirable mass after intervention and the OBC error rate for the mammalian cell cycle network**

**Control gene**	$\boldsymbol {\mathrm {E}_{\Lambda } [\pi _{\mathcal {R}^{\ast }\mathcal {U}}]}$	$\boldsymbol {\hat {\varepsilon } (\psi _{\text {OBC}})}$
E2F	0.2889	0.6572
CycE	0.2334	0.6733
CycA	0.2872	0.6650
Cdc20	0.3386	0.6799
Cdh1	0.3371	0.6704
UbcH10	0.3497	0.6705
CycB	0.2941	0.6631

#### Stress response network

We next consider a p53 signaling pathway derived from the KEGG database [25]. p53 is the tumor suppressor protein encoded by the TP53 gene in humans. p53 activation plays a crucial role in cellular responses to various stress signals that might cause genome instability. These responses include a transient cell cycle arrest, senescence, and apoptosis. The original p53 network involves many genes or proteins, which makes it impossible for us to analyze. Therefore, we only focus on the genes upstream of p53 in its regulatory pathway and construct a BNp based on the relationships listed in Table 3. The network has nine nodes: DNAdamage, p53, p14ARF, ATR, ATM, CHEK1, CHEK2, MDM2, and MDMX. We assume that the states for which DNAdamage and p53 are active and inactive, respectively, are undesirable.

**Table 3:** **Regulatory relationships of a p53 signaling network**

**Gene/protein/signal**	**Predictors: regulatory type (+ / −)**
DNAdamage	DNAdamage: +
p53	ATR: +, CHEK1: +, CHEK2: +,
	MDM2: −, MDMX: −
p14ARF	p14ARF: +
ATR	DNAdamage: +
ATM	DNAdamage: +
CHEK1	ATR: +
CHEK2	ATM: +
MDM2	p14ARF: −, MDMX: +
MDMX	MDM2: −

We construct a BNp following the majority-voting updating rule with *p*=0.01. We then enumerate all the networks that have no greater than one edge removed from or added to the nominal p53 network and use the same criterion as in the mammalian cell cycle example to select networks for inclusion in the uncertainty set of mutated networks. We also calculate the distribution *Λ* assuming a mutation probability of *γ*=0.5. Each node in the network, except DNAdamage and p53, is allowed as a control gene. Given *Λ*, *Θ*, a control gene, and the controlled networks, we partition *Θ* and design an OBC. For this network, $\pi _{\mathcal {R}^{H} \mathcal {U}} = 0.0057$, $\mathrm {E}_{\Lambda } [\!\pi _{\mathcal {R}\mathcal {U}}] = 0.0183$, and *Θ* consists of 829 mutated networks. The classification results are shown in Table 4 for each control gene. Although the uncertainty set is much larger, classification error rates are better than observed for the cell cycle network. The best classification performance is achieved when ATR is the control gene.

**Table 4:** **The expected undesirable mass after intervention and the OBC error rate for the stress response network**

**Control gene**	$\boldsymbol {\mathrm {E}_{\Lambda } [\pi _{\mathcal {R}^{\ast }\mathcal {U}}]}$	$\boldsymbol {\hat {\varepsilon } (\psi _{\text {OBC}})}$
p14ARF	0.0095	0.5175
ATR	0.0104	0.4789
ATM	0.0150	0.5935
CHEK1	0.0110	0.5218
CHEK2	0.0134	0.5561
MDM2	0.00666	0.5376
MDMX	0.0084	0.5220

## Conclusion

We have outlined a framework in which it is possible to utilize prior knowledge regarding cell regulation, for instance pathway information in healthy and aberrant networks, to optimally predict prognosis. That being said, there are several important generalizations of our model that merit further study: (1) integrating partial ignorance of the healthy network itself into our uncertainty class of networks, (2) allowing the network to change over time, thereby taking into account the progressive deterioration of cancer as mutations accumulate, (3) modeling uncertainty in the ideal drug regimen for each network, (4) integrating different types of observations into the analysis, and (5) combining optimal prognosis prediction with optimal treatment recommendations under network uncertainty.

While *ψ*_OBC_ makes optimal prognosis predictions under network uncertainty, obtaining the GAP or any other relevant information from a patient has the effect of reducing uncertainty. A key point in this work is that we study performance with respect to prognosis only. Although one must overcome network uncertainty, it is not necessary to be able to actually infer the network or any mutations, rather, for our purposes one only needs enough relevant data to make good predictions regarding prognosis. Thus, a second major question we address is whether it is possible to successfully predict prognosis with a relatively small amount of data and available biological knowledge.

The larger the uncertainty class, generally the more difficult prognosis becomes. This is an intuitive result: more uncertainty requires more information to draw accurate conclusions. Furthermore, prognosis performance depends on many factors, including the type of cancer (the original healthy network and its associated uncertainty class), the individual patient’s network, and the particular sample drawn from the patient. Very often, prognosis prediction from a single GAP is highly unreliable, even in this optimistic setting with perfect knowledge of healthy biological processes and ideal treatment decisions. In this case, the remedy is to collect more data, for instance time-series GAP measurements, to help identify the patient’s network or at least ensure reliable prognosis prediction. One may be lucky and find that their condition is quite clear from a single measurement, but, at least in our examples, it is typical to find that very little is revealed about one’s condition, necessitating additional lab tests.
